# Squeezed-light-driven force detection with an optomechanical cavity in a Mach–Zehnder interferometer

**DOI:** 10.1038/s41598-020-74629-1

**Published:** 2020-10-15

**Authors:** Chang-Woo Lee, Jae Hoon Lee, Hyojun Seok

**Affiliations:** 1 Department of Physics Education, Kongju National University, Gongju, 32588 South Korea; 2 Korea Research Institute of Standards and Science, Daejeon, 34113 South Korea

**Keywords:** Quantum metrology, Theoretical physics, Quantum optics

## Abstract

We analyze the performance of a force detector based on balanced measurements with a Mach–Zehnder interferometer incorporating a standard optomechanical cavity. The system is driven by a coherent superposition of coherent light and squeezed vacuum field, providing quantum correlation along with optical coherence in order to enhance the measurement sensitivity beyond the standard quantum limit. We analytically find the optimal measurement strength, squeezing direction, and squeezing strength at which the symmetrized power spectral density for the measurement noise is minimized below the standard quantum limit. This force detection scheme based on a balanced Mach–Zehnder interferometer provides better sensitivity compared to that based on balanced homodyne detection with a local oscillator in the low frequency regime.

## Introduction

Quantum mechanics hinders the ability to measure a physical observable with arbitrarily high precision in continuous measurements. The corresponding quantum object being measured is not only agitated by its environment, but also disturbed by a quantum probe^1^. Therefore, the quantum limit of the measurement sensitivity is associated with the quantum characteristics of the object, the probe, and their mutual interactions. Efforts to optically measure the mechanical motion with high sensitivity triggered the research field of cavity optomechanics^2,3^ in which the intensity of a cavity field is coupled to the motion of a mechanical oscillator via radiation pressure force^4,5^. This type of optomechanical measurement first became relevant in research where feeble forces act on macroscopic quantum objects^6^ and in gravitational wave detection^7^. It has been known that the noise resulting from individual photons randomly arriving at the detectors for absorption, and the noise due to random scattering events between intracavity photons and the mechanical oscillator limit the measurement sensitivity of the displacement or the external force^6,8,9^. These two types of noises, namely the photon counting noise and the radiation pressure backaction noise, have opposite scaling behavior with respect to the input laser power: increasing the input power decreases the photon counting noise but increases the radiation pressure backaction noise, and vice versa. Balancing these two noises gives rise to the so-called standard quantum limit (SQL) that describes the limit of sensitivity for conventional interferometric measurements^6,8,9^. Routes to enhancing the sensitivity beyond the SQL thus requires evading the photon counting noise or the radiation pressure backaction noise.

Nonclassical states of light are possible resources for enhancing the sensitivity of displacement or force sensors. Particularly, squeezed states of light are one of the most prominent nonclassical probes of which the quantum fluctuations in one of the quadratures are less than the vacuum noise level^10,11^. Employing squeezed states of light was first proposed to demonstrate a reduction of the photon counting noise in the context of gravitational wave detection, yet undesirably accompanying an increase in the radiation pressure backaction noise^12^. This proposal prompted much theoretical effort on overcoming the SQL for gravitational wave detection, for example, exploiting quantum correlation in the input field^13^, frequency-dependent squeezing of the input field^14–17^, and variational measurements of the output field^18–20^. Such suppression of photon counting noise by squeezing has been realized in various types of table-top interferometers including Mach–Zehnder^21^, polarization^22^, Sagnac^23^, and Michelson interferometers^24^ and recently demonstrated with gravitational wave interferometers^25^. It has also been experimentally shown that squeezing the input field can modify the radiation pressure backaction noise^26^.

Another approach to surpassing the SQL is using quantum nondemolition (QND) measurements in which the observable being measured is not dynamically coupled to its conjugate observable^27–29^. In QND measurements, such dynamical decoupling restrains backaction noise of the conjugate observable from being fed back into the observable being measured, allowing for backaction-evading measurements. A simple example of QND measurements in cavity optomechanics is to measure a static observable, for instance, only one of the quadratures of the mechanical motion^27–30^. Such backaction-evading measurements have been experimentally demonstrated by modulating the amplitude of the driving field in single-mode mechanical systems^31–34^ and in a collective quadrature of multimode mechanical systems^35,36^. Recently, dynamical decoupling between an observable and its conjugate was proposed in the so-called quantum–mechanics-free subsystem (QMFS) by introducing an anti-backaction noise path in the system dynamics for coherent cancellation of the backaction noise^37,38^. Such observables in the QMFS are dynamical QND observables in that they evolve in time but are not coupled to their conjugate observables^38^. Backaction-evading measurements of motion based on QMFS have been proposed in a variety of optomechanical systems including an optomechanical cavity incorporating Kerr medium^39^, Bose–Einstein condensates^40^, an optomechanical cavity with an auxiliary cavity^41^, and hybrid atom-optomechanical systems^42,43^. Furthermore, it has recently been reported that the backaction noise is somewhat suppressed in atomic ensembles that are subject to external magnetic fields^44^.

In this paper, we study schemes employing quantum correlations in squeezed states of light and further utilize the notion of coherent quantum-noise cancellation developed in QND measurements to suppress the photon counting noise in an optomechanical force sensor operating in the low frequency regime. Specifically, we analyze the performance of a force detector based on a balanced Mach–Zehnder interferometer (MZI) incorporating a standard optomechanical cavity. The optomechanical system is driven by a coherent superposition of coherent light and squeezed vacuum field, providing quantum correlation along with optical coherence in order to enhance the measurement sensitivity beyond the SQL. We analytically find the optimal measurement strength, squeezing direction, and squeezing strength at which the symmetrized power spectral density (sPSD) for the measurement noise is minimized below the SQL. This force detection scheme based on a balanced MZI provides better sensitivity compared to that based on a balanced homodyne detection with a local oscillator field in the low frequency regime.

The remainder of this paper is structured as follows. In “Results” section we provide a description of the model system of interest and discuss the quantum characteristics of the optomechanical cavity output field. We then compare the measurement sensitivity of the two detection schemes, specifically, the balanced homodyne detection scheme and the balanced MZI scheme. In “Methods” section we present detailed derivation of the sPSD of the measurement noise, and subsequently show the optimization processes for suppressing the sPSD with respect to the squeezing direction and squeezing strength. Finally, we give our summary and conclusions in “Conclusions” section.

## Results

### Model system

**Figure 1:**
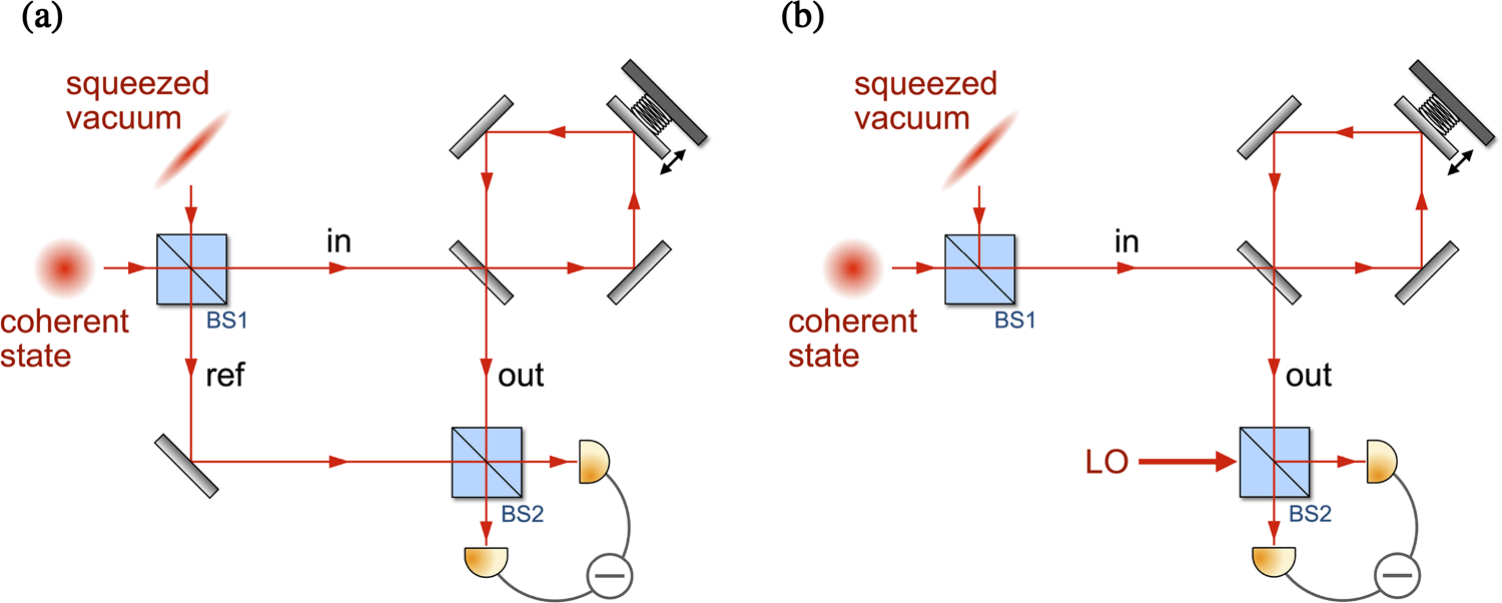
(**a**) Schematics of the Mach–Zehnder interferometer incorporating an optomechanical cavity for quantum enhanced force detection. The coherent field and squeezed vacuum field are combined at the first 50/50 beam splitter (BS1) producing an input field for the optomechanical cavity and a correlated reference field. The field exiting the optomechanical cavity is then combined with the reference beam at the second 50/50 beam splitter (BS2) for balanced detection. (**b**) Schematics of a standard homodyne optomechanical force sensing setup. The output field of the optomechanical cavity is combined with a local oscillator field at BS2. In both schemes, the photon flux difference between the two output ports of BS2 is measured in order to analyze the noise spectrum.

We consider a Mach–Zehnder interferometer in which an optomechanical cavity of resonant frequency $\omega _c$ with a harmonically bound mirror of mass *m* and mechanical frequency $\Omega$ is integrated into one of the optical paths depicted in Fig. 1a. A coherent mixture of monochromatic coherent light and squeezed vacuum is created with a 50/50 beam splitter (BS1). The coherent light oscillates at frequency $\omega _L$ while the broadband squeezed vacuum is characterized by squeezing strength *r*, squeezing direction $\theta$, and central frequency $\omega _L$. The optomechanical system is optically driven by this coherently superposed field, and the output field from the optomechanical cavity is combined with the reference field at a second 50/50 beam splitter (BS2). In order to analyze the quantum noise introduced by this measurement scheme, the difference in photon flux between the output ports of BS2 is measured, i.e., balanced detection. The Hamiltonian governing the dynamics of the optomechanical system in a frame rotating at the driving frequency reads

1 $$\begin{aligned} {\hat{H}} = -\hbar \Delta {\hat{a}}^\dagger {\hat{a}}+i\hbar \sqrt{\kappa _{\mathrm{in}}}\alpha _{\mathrm{in}}({\hat{a}}^\dagger -{\hat{a}})+\frac{\hbar \Omega }{2}({\hat{q}}^2+{\hat{p}}^2)-\hbar g_0\sqrt{2}{\hat{a}}^\dagger {\hat{a}}{\hat{q}}+{\hat{H}}_{\mathrm{diss}} + {\hat{H}}_{\mathrm{ext}}. \end{aligned}$$

The first two terms describe the cavity field driven by a pump field of detuning $\Delta = \omega _L - \omega _c$ with rate $\sqrt{\kappa _{\mathrm{in}}}\alpha _{\mathrm{in}}$ where $\kappa _{\mathrm{in}}$ is the loss rate through the cavity input port mirror, and $\alpha _{\mathrm{in}}=\sqrt{{{\mathcal {P}}}/(\hbar \omega _L)}$ is the coherent amplitude of the input field with ${{\mathcal {P}}}$ being the continuous wave optical power into the cavity. The bosonic annihilation operator of the cavity field ${\hat{a}}$ satisfies the relation $[{\hat{a}}, {\hat{a}}^{\dag }]=1$. The third and fourth terms describe the mechanical oscillator driven by the intracavity radiation pressure, where $g_0$ is the single-photon optomechanical coupling coefficient. The position and momentum operators of the mechanical oscillator are normalized by the natural length $q_0=\sqrt{\hbar /(m\Omega )}$ and momentum $p_0= \sqrt{\hbar m\Omega }$ so that the dimensionless position and momentum operators of the mechanics ${\hat{q}}$ and ${\hat{p}}$ satisfy $[{\hat{q}}, {\hat{p}}]=i$. ${\hat{H}}_{\mathrm{diss}}$ which accounts for dissipation and decoherence represents the incoherent interaction of the optomechanical system with the optical reservoir at zero temperature and the mechanical bath at finite temperature *T*. Finally, ${\hat{H}}_{\mathrm{ext}}$ describes the coupling between the momentum of the mechanical oscillator and an external force to be measured. In this setup, the external force changes the dynamic variables for the mechanics, and subsequently the phase of the output optical field through the optomechanical interaction. In this manuscript, we assume a weak external force where the effects on the optomechanical system is similar to other noise levels. Hence, we focus only on the generic quantum noise generated by the force measurement process.

### Output field from the optomechanical cavity

#### Mean fields

The semiclassical equation of motion for the cavity field derived from Eq. (1) can be written as

2 $$\begin{aligned} {\dot{\alpha }} = (i\Delta _{\mathrm{eff}}-\kappa /2)\alpha +\sqrt{\kappa _{\mathrm{in}}}\alpha _{\mathrm{in}}, \end{aligned}$$

where the classical mean field amplitude for the cavity field is denoted by $\alpha =\langle {\hat{a}}\rangle$ and the effective detuning is defined by $\Delta _{\mathrm{eff}} = \Delta +\sqrt{2}g_0\langle q\rangle$. We assume for simplicity that there are no other loss channels except that from the cavity input port. Therefore, the total dissipation rate for the cavity field is simply the loss rate of the input port mirror, $\kappa = \kappa _{\mathrm{in}}$. In the case where there are other loss channels, such as cavity internal loss, one can expect limited quantum enhancement of the measurement sensitivity^45,46^. Then the steady-state solution for the mean cavity field amplitude in the long-time limit can be obtained as

3 $$\begin{aligned} \alpha _{\mathrm{ss}} = \frac{\sqrt{\kappa }}{-i\Delta _{\mathrm{eff}}+\kappa /2}\alpha _{\mathrm{in}}. \end{aligned}$$

The steady-state position for the mechanics can be similarly obtained as $\langle q\rangle _{\mathrm{ss}}= -\sqrt{2}g_0|\alpha _{\mathrm{ss}}|^2/\Omega$. Invoking the input-output relation, ${\hat{a}}_{\mathrm{out}}= {\hat{a}}_{\mathrm{in}}-\sqrt{\kappa _{\mathrm{in}}}{\hat{a}}$, results in the steady-state coherent amplitude for the cavity output field as

4 $$\begin{aligned} \alpha _{\mathrm{out}} = \frac{-i\Delta _{\mathrm{eff}}-\kappa /2}{-i\Delta _{\mathrm{eff}}+\kappa /2}\alpha _{\mathrm{in}}. \end{aligned}$$

Notice that the magnitude of the coherent amplitude is the same for the cavity input and output fields, but there is a difference in phase depending on the optomechanical interaction. This phase shift of the optical field accounts for various static optomechanical effects, for example, the static optomechanical spring effect and the optical bistability^47,48^. In this paper, we assume that the effective detuning is zero so that the steady-state intracavity field amplitude is simply $\alpha _{\mathrm{ss}} = 2\alpha _{\mathrm{in}}/\sqrt{\kappa }$ and the classical amplitude of the input and output field is $180^\circ$ out of phase, $\alpha _{\mathrm{out}}= -\alpha _{\mathrm{in}}$. As is well-known, zero effective detuning does not lead to various dynamic optomechanical effects including optomechanical cooling or amplification of the mechanical oscillator^49–51^, entanglement between the mechanical motion and cavity fields^52^, and optomechanically induced transparency^53^, to mention just a few.

#### Quantum fluctuations

So as to better capture the steady-state features of the quantum fluctuations in the optical and mechanical fields, we transform to a displaced frame where the expectation values of the optical field operator and the mechanical oscillator position operator are zero. Performing the displacement operation, ${\hat{a}} \rightarrow \alpha _{\mathrm{ss}}+{\hat{d}}$, ${\hat{q}} \rightarrow \langle q\rangle _{\mathrm{ss}}+{\hat{Q}}$, the Heisenberg–Langevin equations of motion for the quantum fluctuations of the optical field and the mechanical oscillator can be approximated as^46^

5a $$\begin{aligned} \dot{{\hat{X}}}&= -\frac{\kappa }{2}{\hat{X}} +\sqrt{\kappa }{\hat{X}}_{\mathrm{in}}, \end{aligned}$$

5b $$\begin{aligned} \dot{{\hat{Y}}}&= -\frac{\kappa }{2}{\hat{Y}} +2g{\hat{Q}}+\sqrt{\kappa }{\hat{Y}}_{\mathrm{in}}, \end{aligned}$$

5c $$\begin{aligned} \dot{{\hat{Q}}}&= \Omega {\hat{P}}, \end{aligned}$$

5d $$\begin{aligned} \dot{{\hat{P}}}&= -\Omega {\hat{Q}}-\Gamma {\hat{P}}+2g{\hat{X}}+\sqrt{2\Gamma }{\hat{P}}_{\mathrm{in}}, \end{aligned}$$

where the intrinsic nonlinear optomechanical coupling is neglected in the weak coupling regime, $g=g_0\alpha _{\mathrm{ss}}$ is the field-amplified optomechanical coupling strength, and $\Gamma$ is the mechanical dissipation rate. The operators ${\hat{X}}=\frac{1}{\sqrt{2}}({\hat{d}}^\dag +{\hat{d}})$ and ${\hat{Y}}=\frac{i}{\sqrt{2}}({\hat{d}}^\dag -{\hat{d}})$ represent the quantum fluctuations of the amplitude and phase quadrature for the intracavity field, and ${\hat{X}}_{\mathrm{in}}$ and ${\hat{Y}}_{\mathrm{in}}$ are the input field noise operators for the two quadratures of the cavity field. Finally, ${\hat{P}}_{\mathrm{in}}={\hat{F}}_{\mathrm{th}}+{\hat{F}}_{\mathrm{ext}}$ represents the force acting on the mechanical oscillator where ${\hat{F}}_{\mathrm{th}}$ and ${\hat{F}}_{\mathrm{ext}}$ are the operators for the thermal force and the external force of interest, i.e., force to be detected, with units of $\sqrt{\mathrm{Hz}}$.

Now that the Heisenberg equations of motion for the quantum fluctuations are linear with respect to the field operators, it is convenient to solve them in the frequency domain exploiting the Fourier transform for the operators,

6 $$\begin{aligned} {\hat{O}}(\omega )=\frac{1}{\sqrt{2\pi }}\int dt~e^{i\omega t} {\hat{O}}(t). \end{aligned}$$

With the input-output relation in the frequency domain, ${\hat{d}}_{\mathrm{out}}(\omega )= {\hat{d}}_{\mathrm{in}}(\omega )-\sqrt{\kappa }{\hat{d}}(\omega )$, the fluctuations in the quadrature for the output field are obtained as

7 $$\begin{aligned} {\hat{X}}_{\mathrm{out}}(\omega )= & {} -\frac{\chi _o^{*}}{\chi _o}{\hat{X}}_{\mathrm{in}}(\omega ), \end{aligned}$$

8 $$\begin{aligned} {\hat{Y}}_{\mathrm{out}}(\omega )= & {} -\frac{\chi _o^{*}}{\chi _o}{\hat{Y}}_{\mathrm{in}}(\omega ) +4G\chi _o^{*2}\chi _m{\hat{X}}_{\mathrm{in}}(\omega )-2\sqrt{2\Gamma G}\chi _o^{*}\chi _m{\hat{P}}_{\mathrm{in}}(\omega ). \end{aligned}$$

Here, we have defined the measurement strength

9 $$\begin{aligned} G=g^2\kappa =4g_0^2{{\mathcal {P}}}/(\hbar \omega _L), \end{aligned}$$

where we used the definition of the amplified optomechanical strength $g=g_0\alpha _{\mathrm{ss}}$, the steady-state intracavity field amplitude $\alpha _{\mathrm{ss}}=2\alpha _{\mathrm{in}}/\kappa$ for resonant driving, and the coherent amplitude of the input field $\alpha _{\mathrm{in}}=\sqrt{{{\mathcal {P}}}/(\hbar \omega _L)}$. Note that while the intracavity photon number is inversely proportional to the cavity linewidth, the measurement strength *G* is independent of the cavity linewidth since it is directly related to the photon number in the output field. The optical and mechanical susceptibilities are given by

10 $$\begin{aligned} \chi _o= & {} \frac{1}{i\omega +\kappa /2}, \end{aligned}$$

11 $$\begin{aligned} \chi _m= & {} \frac{\Omega }{\Omega ^2-\omega ^2-i\omega \Gamma }. \end{aligned}$$

As can be inferred from Fig. 1, the quantum noise in the input field of the optomechanical system derives from the coherent superposition of the vacuum fluctuations ${\hat{d}}_{\mathrm{v}}$ and squeezed vacuum fluctuations ${\hat{d}}_{\mathrm{sq}}$. Since the squeezed vacuum undergoes a reflection at BS1 there is a relative phase of $90^\circ$ between the two fields, i.e., ${\hat{d}}_{\mathrm{in}}=({\hat{d}}_{\mathrm{v}}+i{\hat{d}}_{\mathrm{sq}})/\sqrt{2}$. The quantum fluctuations in the quadrature of the output field then become

12 $$\begin{aligned} {\hat{X}}_{\mathrm{out}}(\omega )= & {} -\frac{1}{\sqrt{2}}\frac{\chi _o^{*}}{\chi _o}\left[ {\hat{X}}_{\mathrm{v}}(\omega )-{\hat{Y}}_{\mathrm{sq}}(\omega )\right] , \end{aligned}$$

13 $$\begin{aligned} {\hat{Y}}_{\mathrm{out}}(\omega )= & {} -\frac{1}{\sqrt{2}}\frac{\chi _o^{*}}{\chi _o}\left[ {\hat{Y}}_{\mathrm{v}}(\omega )+{\hat{X}}_{\mathrm{sq}}(\omega )\right] +\frac{1}{\sqrt{2}}4G\chi _o^{*2}\chi _m \left[ {\hat{X}}_{\mathrm{v}}(\omega )-{\hat{Y}}_{\mathrm{sq}}(\omega )\right] -2\sqrt{2\Gamma G}\chi _o^{*}\chi _m{\hat{P}}_{\mathrm{in}}(\omega ). \end{aligned}$$

It is important to note that the external force is imprinted only on the phase quadrature of the output field. This consequence can be attributed to having zero effective detuning which leads to the amplitude quadrature of the output field containing no information of the mechanics^54^. Thus, in order to capture information with regard to the external forces of interest one must measure ${\hat{Y}}_{\mathrm{out}}(\omega )$. The additional quantum fluctuations are inevitably introduced in the measurement of the phase quadrature of the output field.

### Balanced homodyne detection with a local oscillator field

We first explore a scheme based on a balanced homodyne detection with a local oscillator (LO) field which may be considered more prototypical, as depicted in Fig. 1b in order to better appreciate the performance of our proposed balanced Mach–Zehnder measurement scheme which optimally utilizes quantum interference effects. As shown in Fig. 1b, the output field from the optomechanical cavity is coherently mixed with a LO field at BS2. The optical fields ${\hat{a}}_{+},~{\hat{a}}_{-}$ at the two output ports of BS2 can be described as

14 $$\begin{aligned} \begin{pmatrix} {\hat{a}}_{+} \\ {\hat{a}}_{-} \end{pmatrix} =\frac{1}{\sqrt{2}} \begin{pmatrix} 1 &{} i \\ i &{} 1 \end{pmatrix} \begin{pmatrix} {\hat{a}}_{\mathrm{out}} \\ {\hat{a}}_{\mathrm{LO}} \end{pmatrix}, \end{aligned}$$

where ${\hat{a}}_{\mathrm{LO}}$ is the bosonic annihilation operator for the LO field. If the amplitude of the LO field is sufficiently larger than that of the cavity output field, the LO field can be treated classically. The quantum fluctuations in the photon flux difference between the two output ports ${\hat{I}} ={\hat{a}}_{-}^\dagger {\hat{a}}_{-}-{\hat{a}}_{+}^\dagger {\hat{a}}_{+}$, can now be approximated as

15 $$\begin{aligned} \delta {\hat{I}} = -\sqrt{2}|\alpha _{\mathrm{LO}}|({\hat{Y}}_{\mathrm{out}}\cos \varphi +{\hat{X}}_{\mathrm{out}}\sin \varphi ), \end{aligned}$$

where $\alpha _{\mathrm{LO}}= |\alpha _{\mathrm{LO}}|e^{i \varphi }$ is the complex amplitude of the LO field. Since the force we want to detect is only dependent on ${\hat{Y}}_{\mathrm{out}}$, see Eq. (13), setting $\varphi =\pi$ for maximum amount of information gives rise to

16 $$\begin{aligned} \delta {\hat{I}} = \sqrt{2}|\alpha _{\mathrm{LO}}|{\hat{Y}}_{\mathrm{out}}. \end{aligned}$$

It should be kept in mind that $\delta {\hat{I}}$ does not contain interference between ${\hat{Y}}_{\mathrm{out}}$ and additional quantum fluctuations since the LO is treated classically. The role of the LO field in this scheme is to choose the appropriate quadrature for measuring the output field, ${\hat{Y}}_{\mathrm{out}}$, without adding additional quantum fluctuations.

In this situation, the unbiased estimator for the external force of interest reads

17 $$\begin{aligned} {\hat{F}}_{\mathrm{est}}(\omega ) = -\frac{\delta I(\omega )}{4|\alpha _{\mathrm{LO}}|\sqrt{\Gamma G}\chi _o^{*}\chi _m} ={\hat{F}}(\omega )+{\hat{F}}_{\mathrm{ext}}(\omega ), \end{aligned}$$

where ${\hat{F}}(\omega )$ is the quantum noise introduced in this force measurement scheme and can be written as

18 $$\begin{aligned} {\hat{F}}(\omega ) = c_{Y_{\mathrm{v}}}{\hat{Y}}_{\mathrm{v}}(\omega )+c_{X_{\mathrm{sq}}}{\hat{X}}_{\mathrm{sq}}(\omega )+c_{X_{\mathrm{v}}}{\hat{X}}_{\mathrm{v}}(\omega )+c_{Y_{\mathrm{sq}}}{\hat{Y}}_{\mathrm{sq}}(\omega )+ {\hat{F}}_{\mathrm{th}}(\omega ) \end{aligned}$$

with

19 $$\begin{aligned} c_{Y_{\mathrm{v}}} = \frac{1}{4\sqrt{\Gamma G}\chi _o\chi _m},\quad c_{X_{\mathrm{sq}}} = c_{Y_{\mathrm{v}}}, \quad c_{X_{\mathrm{v}}} = -\sqrt{\frac{G}{\Gamma }}\chi _o^{*},\quad c_{Y_{\mathrm{sq}}}=-c_{X_{\mathrm{v}}}. \end{aligned}$$

It is apparent from Eq. (19) that the quantum fluctuations in the the phase quadrature of the vacuum field are in phase with those in the amplitude quadrature of the squeezed vacuum field. Contrarily, the quantum fluctuations in the amplitude quadrature of the vacuum field are perfectly out of phase with those in the phase quadrature of the squeezed vacuum field. Additionally, we can deduce that in the weak-driving regime the quantum noise in the phase quadrature for the vacuum field and the amplitude quadrature for the squeezed vacuum field will be the dominant sources of detection noise, since $c_{Y_{\mathrm{v}}}$ and $c_{X_{\mathrm{sq}}}$ are inversely proportional to the square root of the measurement strength. On the other hand, since both $c_{X_{\mathrm{v}}}$ and $c_{Y_{\mathrm{sq}}}$ are proportional to the square root of the measurement strength, the quantum noises in the amplitude quadrature for the vacuum field and the phase quadrature for the squeezed vacuum field will be the dominant sources of detection noise in the strong-driving regime. Finally, the thermal fluctuations of the mechanical oscillator are independent of the measurement strength, as expected.

We are now in a position to calculate the sPSD of ${\hat{F}}(\omega )$ relevant to the homodyne detection

20 $$\begin{aligned} \bar{S}_{FF}(\omega ) =\frac{1}{2}\left[ S_{FF}(\omega )+S_{FF}(-\omega )\right] , \end{aligned}$$

where $S_{FF}(\omega )$ is the self-PSD of ${\hat{F}}(\omega )$ and is defined by

21 $$\begin{aligned} S_{FF}(\omega )=~ \int _{-\infty }^{\infty } d\omega '\langle {\hat{F}}^\dag (-\omega '){\hat{F}}(\omega )\rangle . \end{aligned}$$

Since ${\hat{F}}(\omega )$ has the dimension of $1/\sqrt{\mathrm{Hz}}$, $\bar{S}_{FF}(\omega )$ is thus dimensionless in the unit system used in this manuscript. Note that $2\Gamma m\hbar \Omega \bar{S}_{FF}(\omega )$ with units of newtons squared per hertz should be used when comparing with experimental measurements in the laboratory. Assuming that the vacuum fluctuations, the squeezed vacuum fluctuations, and the thermal fluctuations of the mechanics have no memory effect and are uncorrelated with respect to each other, the derivation of the sPSD of ${\hat{F}}(\omega )$ is rather straightforward and is presented in “Methods” section. The resulting sPSD of ${\hat{F}}(\omega )$ can be decomposed into contributions from the vacuum, squeezed vacuum, and thermal fluctuations as

22 $$\begin{aligned} \bar{S}_{FF}(\omega ) = \bar{S}_{\mathrm{v}}(\omega ) +\bar{S}_{\mathrm{sq}}(\omega ) + \bar{S}_{\mathrm{th}}(\omega ), \end{aligned}$$

where the contribution from the vacuum noise is

23 $$\begin{aligned} \bar{S}_{\mathrm{v}}(\omega ) = \frac{1}{2}\left[ |c_{X_{\mathrm{v}}}|^2+|c_{Y_{\mathrm{v}}}|^2\right] , \end{aligned}$$

the contribution from the squeezed vacuum noise

24 $$\begin{aligned} \bar{S}_{\mathrm{sq}}(\omega ) = \frac{1}{2} \cosh (2r)\left[ |c_{X_{\mathrm{sq}}}|^2+|c_{Y_{\mathrm{sq}}}|^2 \right] +\frac{1}{2}\sinh (2r)\left[ \cos \theta \left( |c_{X_{\mathrm{sq}}}|^2-|c_{Y_{\mathrm{sq}}}|^2\right) -\sin \theta ~\mathrm{Re}[2c_{X_{\mathrm{sq}}}c_{Y_{\mathrm{sq}}}^*]\right] , \end{aligned}$$

and the contribution from thermal fluctuations of the mechanics

25 $$\begin{aligned} \bar{S}_{\mathrm{th}}(\omega ) = \frac{\omega }{\Omega }\left[ {\bar{n}}_{\mathrm{th}}(\omega )+\frac{1}{2}\right] , \end{aligned}$$

with ${\bar{n}}_{\mathrm{th}}(\omega )= [\exp (\hbar \omega /k_BT)-1]^{-1}$ being the mean phonon occupation number in thermal equilibrium with the mechanical reservoir at finite temperature *T*.

#### Standard quantum limit

**Figure 2:**
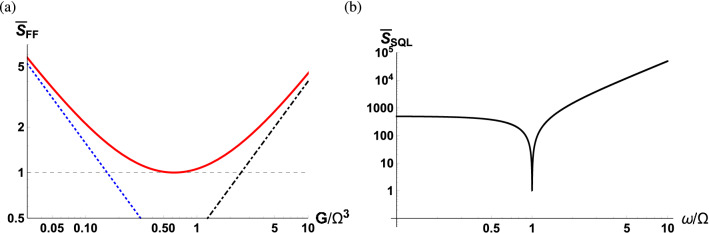
(**a**) sPSD of ${\hat{F}}(\omega )$ as a function of scaled measurement strength $G/\Omega ^3$ (red solid curve) along with the photon counting noise (blue dotted curve), radiation pressure backaction noise (black dot-dashed curve) and the standard quantum limit (gray dashed curve). (**b**) Standard quantum limit as a function of the scaled detection frequency. The parameters used are $\kappa /\Omega = 10^2$, $\Gamma /\Omega = 10^{-3}$.

In the case when the squeezing strength is zero, $\bar{S}_{FF}(\omega )$ is given by

26 $$\begin{aligned} \bar{S}_{FF}(\omega ) = \frac{1}{16\Gamma G|\chi _o|^2|\chi _m|^2}+\frac{G |\chi _o|^2}{\Gamma } +\frac{\omega }{\Omega }\left[ {\bar{n}}_{\mathrm{th}}(\omega )+\frac{1}{2}\right] . \end{aligned}$$

The first term, stemming from $Y_{\mathrm{v}}(\omega )$ and $X_{\mathrm{sq}}(\omega )$, is known as the photon counting noise. The second term is known as the radiation pressure backaction noise and derives from $X_{\mathrm{v}}(\omega )$ and $Y_{\mathrm{sq}}(\omega )$. Notice that the optical susceptibility affects the photon counting noise and the radiation pressure backaction noise in an inversed manner. Roughly speaking, a large cavity linewidth decreases the intracavity photon number, and therefore increases the photon counting noise while decreasing the radiation pressure backaction noise. Figure 2a shows the sPSD of ${\hat{F}}(\omega )$ as a function of the scaled measurement strength at $\omega =\Omega$. This plot depicts the common behavior that the photon counting noise is the dominant source of fluctuations at weak measurement strengths while the backaction noise becomes dominant at strong measurement strengths. For this reason, the sPSD can be minimized with respect to the measurement strength where one balances between the photon counting noise and radiation pressure backaction noise to reach the SQL

27 $$\begin{aligned} \bar{S}_{\mathrm{SQL}}(\omega ) = \frac{1}{2\Gamma |\chi _m|}+\frac{\omega }{2\Omega }, \end{aligned}$$

with the mechanical bath at zero temperature and the measurement strength

28 $$\begin{aligned} G_{\mathrm{SQL}}= \frac{1}{4|\chi _o|^2|\chi _m|}. \end{aligned}$$

The first term in Eq. (27) comes from the quantum nature of the optical field and the second term results from the zero-point fluctuations of the mechanics. It is interesting to point out that the radiation pressure backaction gives rise to the dependence of the response function of the mechanics in the first term. Figure 2b shows the SQL as a function of the scaled detection frequency $\omega /\Omega$ with a fixed mechanical damping rate. Since the mechanical susceptibility is maximized at the mechanical frequency, $\chi _m(\Omega )=i/\Gamma$, the sPSD for the measurement noise reaches the lower bound which is unity in the unit system used in this manuscript.

#### Beating the SQL with squeezed vacuum

In the case where $r\ne 0$, one can exploit the quantum correlation in the squeezed vacuum fluctuations to increase the sensitivity beyond the SQL. It is obvious from Eq. (24) that the sPSD resulting from the squeezed vacuum fluctuations has two contributions. The first term in Eq. (24) represents the photon counting noise and the radiation pressure backaction noise which increase with the squeezing strength while are independent of the direction of squeezing. The second term is associated with the quantum correlation of the squeezed vacuum fluctuations and is subject to both the direction and strength of squeezing. Therefore, the sPSD can be minimized with respect to the angle of squeezing as well as the squeezing strength. With broadband and frequency-dependent squeezing^55^, the optimization procedure is rather straightforward and is presented in “Methods”* section. By applying the optimal squeezing strength and squeezing angle at a given frequency, the minimized sPSD can be found as

29 $$\begin{aligned} \bar{S}_{FF}(\omega ) = \frac{1}{32\Gamma G|\chi _o|^2|\chi _m|^2}+\frac{G |\chi _o|^2}{2\Gamma }+\frac{\omega }{4\Omega }+\frac{\omega }{\Omega }\left[ {\bar{n}}_{\mathrm{th}}(\omega )+\frac{1}{2}\right] . \end{aligned}$$

The first and second terms introduced by the vacuum fluctuations characterize the photon counting noise and radiation pressure backaction noise, respectively. Notice that the sPSD due to the photon counting noise and radiation pressure backaction noise are halved compared to those in Eq. (26) since the photon counting and radiation pressure backaction noise in Eq. (29) result from only the vacuum fluctuations. The third term results from the squeezed vacuum fluctuations and is optimized with respect to both the squeezing strength and direction, but surprisingly depends only on the detection frequency scaled to the mechanical frequency. Finally, by balancing the photon counting and backaction noise the sPSD can reach an optimized lower bound

30 $$\begin{aligned} \bar{S}_{FF}^{\mathrm{opt}}(\omega ) = \frac{1}{4\Gamma |\chi _m|}+\frac{3\omega }{4\Omega } \end{aligned}$$

with the mechanical bath at zero temperature, the optimum measurement strength

31 $$\begin{aligned} G_{\mathrm{opt}} = G_{\mathrm{SQL}}, \end{aligned}$$

the optimum squeezing direction

32 $$\begin{aligned} \theta _{\mathrm{opt}} = \frac{\pi }{2}, \end{aligned}$$

and the optimum squeezing strength

33 $$\begin{aligned} r_{\mathrm{opt}} =\frac{1}{2}\mathrm{sech}^{-1}\left[ \frac{\Gamma \omega |\chi _m|}{\Omega }\right] . \end{aligned}$$

Notice that the optimum measurement strength is the same as that for the SQL in that the optimized photon counting and radiation backaction noise scale in the same manner as that for the SQL. In addition, $\theta _{\mathrm{opt}}$ is independent of the physical parameters of the optomechanical system as well as the detection frequency. This underlies the fact that the optimized squeezing is along the $\pi /4$ direction in phase space so as to lead to a maximum negative correlation between quantum fluctuations in the amplitude and phase quadratures^13^. In contrast to the squeezing direction, $r_{\mathrm{opt}}$ relies on mechanical parameters such as the quality factor of the mechanical oscillator and the detection frequency.

**Figure 3:**
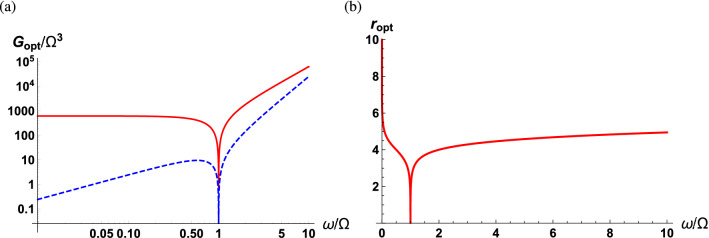
(**a**) Optimal measurement strength as a function of scaled detection frequency $\omega /\Omega$ for the case where the output field interferes with a classical LO field (red solid curve) and a MZI reference field (blue dashed curve). (**b**) Optimized squeezing strength as a function of the scaled detection frequency. The parameters used are $\kappa /\Omega = 10^2$ and $\Gamma /\Omega = 10^{-3}$.

Figure 3a,b show the scaled optimal measurement strength and the optimal squeezing strength as a function of scaled detection frequency $\omega /\Omega$ with a fixed cavity linewidth and mechanical damping rate (red solid curve), respectively. When the detection frequency is the same as the mechanical frequency, the mechanical susceptibility is maximized and both the optimum measurement strength and squeezing strength are minimized. In this on-resonance case, the optimal squeezing strength is zero, indicating that the squeezed vacuum field can not assist in beating the SQL for enhanced sensitivity. As the detection frequency moves off resonant from the mechanical frequency, the mechanical susceptibility decreases so that $G_{\mathrm{opt}}$ and $r_{\mathrm{opt}}$ increase in a monotonic fashion. It should be kept in mind that force measurements of dc fields or frequencies much larger than other system parameters are out of scope in this manuscript since the optimum squeezing strength approaches infinity. This condition breaks the essential assumption for the linearization of the optomechanical interaction, i.e., the amplitudes of the quantum fluctuations are much smaller than the relevant classical expectation values^4,5^.

**Figure 4:**
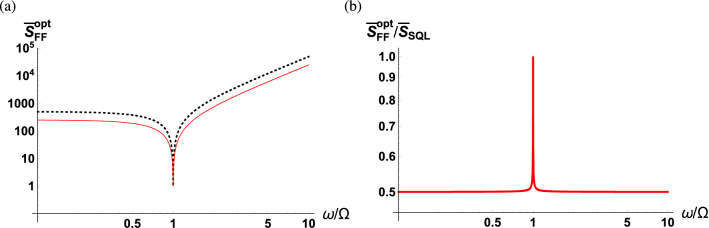
(**a**) Optimum sPSD of ${\hat{F}}(\omega )$ as a function of scaled detection frequency $\omega /\Omega$ (red solid curve) along with the SQL (black dashed curve). (**b**) Optimum sPSD of ${\hat{F}}(\omega )$ normalized to the SQL as a function of the scaled detection frequency. The parameters used are $\Gamma /\Omega = 10^{-3}$.

Figure 4a shows the optimum sPSD along with the SQL as a function of the scaled detection frequency, displaying that the optimum sPSD is always lower than the SQL except on resonance. Figure 4b presents the optimum sPSD normalized with the SQL, indicating that $\bar{S}_{FF}^{\mathrm{opt}}(\omega )$ is one half of the SQL when the detection frequency is far off-resonance. In the case of far off-resonance, the contribution from the squeezed vacuum fluctuations can be considered to be negligible leading to the vacuum field contribution in Eq. (30) to be halved compared to that in Eq. (27).

### Balanced Mach–Zehnder interferometer

In this section, we present the sPSD corresponding to the quantum noise introduced in the measurement process described in Fig. 1a. In this scheme the coherent light experiences a reflection at BS1 where the quantum fluctuations in the reference field can be written as ${\hat{d}}_{\mathrm{ref}}=(i{\hat{d}}_{\mathrm{v}}+{\hat{d}}_{\mathrm{sq}})/\sqrt{2}$. Subsequently, the output field from the optomechanical cavity is coherently mixed with the reference field at BS2. The optical fields ${\hat{a}}_{+},~{\hat{a}}_{-}$ at the two output ports of BS2 can be written as

34 $$\begin{aligned} \begin{pmatrix} {\hat{a}}_{+} \\ {\hat{a}}_{-} \end{pmatrix} =\frac{1}{\sqrt{2}} \begin{pmatrix} 1 &{} i \\ i &{} 1 \end{pmatrix} \begin{pmatrix} {\hat{a}}_{\mathrm{out}} \\ i{\hat{a}}_{\mathrm{ref}} \end{pmatrix}, \end{aligned}$$

where the additional *i* in front of the field operator for the reference field accounts for a reflection at BS1. The quantum fluctuations of the balanced signal between the two output ports ${\hat{I}}$ can be approximated as

35 $$\begin{aligned} \delta {\hat{I}} = \sqrt{2}\alpha _{\mathrm{in}}\left( {\hat{Y}}_{\mathrm{out}}-{\hat{X}}_{\mathrm{ref}}\right) , \end{aligned}$$

where we have used the fact that the reference field is ahead of the input field in relative phase by $90^\circ$, $\alpha _{\mathrm{ref}}=i\alpha _{\mathrm{in}}$, and the output field is $180^\circ$ out of phase relative to the input field, $\alpha _{\mathrm{out}}=-\alpha _{\mathrm{in}}$. ${\hat{X}}_{\mathrm{ref}}=(-{\hat{Y}}_{\mathrm{v}}+{\hat{X}}_{\mathrm{sq}})/\sqrt{2}$ represents the quantum fluctuations in the amplitude quadrature of the reference field. Notice that the quantum fluctuations in the phase quadrature of the output field interfere with those in the amplitude quadrature of the reference field destructively. Owing to the fact that the term for optical shot noise in the phase quadrature of the output field is given by $-\frac{1}{\sqrt{2}}\frac{\chi _o^{*}}{\chi _o}\left( {\hat{Y}}_{\mathrm{v}}+{\hat{X}}_{\mathrm{sq}}\right)$ from Eq. (13), the resulting optical shot noise in $\delta {\hat{I}}$ can be described by $-\alpha _{\mathrm{in}}\chi _o^{*}\left( 2i\omega {\hat{Y}}_{\mathrm{v}}+\kappa {\hat{X}}_{\mathrm{sq}}\right)$. Overall, the unbiased estimator for the external force of interest can be obtained as

36 $$\begin{aligned} {\hat{F}}_{\mathrm{est}}(\omega ) = -\frac{ \delta I(\omega )}{4\alpha _{\mathrm{in}}\sqrt{\Gamma G}\chi _o^{*}\chi _m} \equiv {\hat{F}}(\omega )+{\hat{F}}_{\mathrm{ext}}(\omega ), \end{aligned}$$

where ${\hat{F}}(\omega )$ is the quantum noise concomitant in this force measurement scheme and can be written in the form of Eq. (18) with the relevant coefficients

37 $$\begin{aligned} c_{Y_{\mathrm{v}}} = \frac{i\omega }{2\sqrt{\Gamma G}\chi _m},\quad c_{X_{\mathrm{sq}}} = \frac{\kappa }{2i\omega }c_{Y_{\mathrm{v}}},\quad c_{X_{\mathrm{v}}} = -\sqrt{\frac{G}{{\Gamma }}}\chi _o^{*},\quad c_{Y_{\mathrm{sq}}}=-c_{X_{\mathrm{v}}}. \end{aligned}$$

While $c_{Y_{\mathrm{v}}}= c_{X_{\mathrm{sq}}}$ in Eq. (19), the destructive interference between ${\hat{Y}}_{\mathrm{out}}(\omega )$ and ${\hat{X}}_{\mathrm{ref}}(\omega )$ at BS2 results in a different scaling between $Y_{\mathrm{v}}(\omega )$ and $X_{\mathrm{sq}}(\omega )$. Keep in mind that the contribution from the squeezed vacuum fluctuations manifests itself in the regime where the cavity linewidth is much larger than the detection frequency, therefore, we limit ourselves to the regime where $\omega \ll \kappa$. Comparing this balanced MZI based scheme with the conventional balanced homodyne scheme, we observe that the optical shot noise now displays quantum interference between the cavity output field and the reference field. On the other hand, the coefficients associated with the fluctuations in the amplitude quadrature of the vacuum field and the phase quadrature of the squeezed vacuum field are the same for the two schemes, so that the contribution from the radiation pressure backaction noise and the thermal noise remain the same. In other words, the quantum interference between the cavity output field and the reference field alters only the photon counting noise which differentiates the two schemes.

Before exploiting the quantum correlation in the squeezed vacuum field, it is helpful to consider the case where $r=0$ for completeness. In this case, the sPSD of ${\hat{F}}(\omega )$ is given by

38 $$\begin{aligned} \bar{S}_{FF}(\omega ) = \frac{1}{8\Gamma G|\chi _o|^2|\chi _m|^2}+\frac{G |\chi _o|^2}{\Gamma }+\frac{\omega }{\Omega }\left[ {\bar{n}}_{\mathrm{th}}(\omega )+\frac{1}{2}\right] . \end{aligned}$$

As expected, the radiation pressure backaction noise contribution and thermal noise contribution are the same as those in Eq. (26). However, the photon counting noise is doubled compared to that in Eq. (26) due to the presence of optical shot noise in the reference field. This result entails that we should not expect any additional benefits by exploiting quantum interference in the case of zero squeezing strength.

#### Beating the SQL based on squeezed vacuum and quantum interference

We now turn to the case where $r\ne 0$ to explore the effects of quantum interference and quantum correlation in the squeezed vacuum field on the detected quantum noise. As mentioned before, minimizing the sPSD of ${\hat{F}}(\omega )$ with respect to the squeezing strength and direction leads to

39 $$\begin{aligned} \bar{S}_{FF}(\omega ) = \frac{\omega ^2}{8\Gamma G|\chi _m|^2}+\frac{G |\chi _o|^2}{2\Gamma } +\frac{\omega \kappa |\chi _o|^2}{4\Omega \Gamma }\left| \omega ^2-\Omega ^2-\frac{\Gamma \kappa }{2}\right| +\frac{\omega }{\Omega }\left[ {\bar{n}}_{\mathrm{th}}(\omega )+\frac{1}{2}\right] . \end{aligned}$$

**Figure 5:**
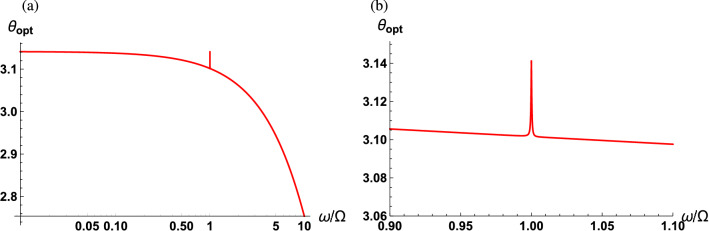
Optimum squeezing direction as a function of the scaled detection frequency at the optimum measurement strength (**a**) across the low frequency regime and (**b**) enlarged in the vicinity of the mechanical resonance. The parameters used are $\kappa /\Omega = 10^2$, $\Gamma /\Omega = 10^{-3}$.

It is instructive to discuss the key features following the quantum interference in Eq. (39), which we note is absent in Eq. (29). First, notice that while the radiation pressure backaction noise and the thermal noise are the same, the photon counting noise is different when compared to Eq. (29). A large cavity linewidth compared to the detection frequency leads to increased photon counting noise and decreased radiation pressure backaction noise in Eq. (29). However, in Eq. (39), a relatively large cavity linewidth decreases the radiation pressure backaction noise but does not alter the photon counting noise. Second, the term due to the squeezed vacuum fluctuations, i.e., third term in Eq. (39), is minimized at frequency

40 $$\begin{aligned} \Omega _s=\sqrt{\Omega ^2+\frac{\Gamma \kappa }{2}}, \end{aligned}$$

whereas in Eq. (29), this term is independent of the optical cavity parameters. As before, balancing between the photon counting noise and radiation pressure backaction noise can suppress the sPSD of ${\hat{F}}(\omega )$ to reach a lower bound

41 $$\begin{aligned} \bar{S}_{FF}^{\mathrm{opt}}(\omega ) = \frac{\omega |\chi _o|}{2\Gamma |\chi _m|} +\frac{\omega \kappa |\chi _o|^2}{4\Omega \Gamma }\left| \omega ^2-\Omega ^2-\frac{\Gamma \kappa }{2}\right| +\frac{\omega }{2\Omega }, \end{aligned}$$

with the mechanical bath at zero temperature, the optimum measurement strength

42 $$\begin{aligned} G_{\mathrm{opt}} =\frac{\omega }{2|\chi _o||\chi _m|}, \end{aligned}$$

the optimum squeezing direction

43 $$\begin{aligned} \theta _{\mathrm{opt}} = \cos ^{-1}\left[ \frac{\omega ^2-\kappa ^2/4}{\left| \omega ^2 \frac{\chi _o^*(\omega )}{\chi _o(\omega )} +\frac{\kappa ^2}{4}\frac{\chi _m^*(\omega )}{\chi _m(\omega )}\right| }\right] , \end{aligned}$$

and the optimum squeezing strength

44 $$\begin{aligned} r_{\mathrm{opt}}=\frac{1}{2}\mathrm{sech}^{-1}\left[ \frac{\omega ^2\kappa |\chi _o|^3 |\chi _m|\left| \omega ^2-\Omega ^2-\frac{\Gamma \kappa }{2}\right| }{\Omega }\right] . \end{aligned}$$

In this scenario, as shown in Fig. 3a, the optimal measurement strength is lower than that for the scheme using a classical LO, indicating that the optimum sPSD will be obtained with weaker input powers. Additionally, the optimal squeezing direction relies on both the detection frequency scaled to the cavity linewidth and the phase difference between the optical and mechanical susceptibilities. The optimal squeezing direction is displayed in Fig. 5 as a function of the scaled detection frequency in the low-frequency regime ($\omega \ll \kappa$). The optimum squeezing angle monotonically decreases from $\pi$ with the detection frequency, except in the vicinity of the mechanical resonance. At resonance the optimum squeezing angle peaks to a value close to $\pi$. In this case, the phase quadrature operator of the squeezed vacuum field is squeezed.

**Figure 6:**
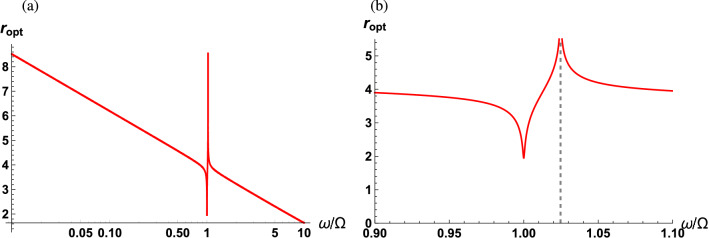
Optimum squeezing strength as a function of the scaled detection frequency at the optimum squeezing direction and measurement strength (**a**) across the low frequency regime and (**b**) enlarged in the vicinity of the mechanical resonance. At $\omega =\Omega _s$, $r_{\mathrm{opt}}$ approaches to infinity (gray dashed line). The parameters used are $\kappa /\Omega = 10^2$, $\Gamma /\Omega = 10^{-3}$.

Figure 6a,b show the optimal squeezing strength as a function of the scaled detection frequency in the low-frequency regime. Figure 6b clearly depicts the locally minimized optimal squeezing strength at $\omega =\Omega$, and that which approaches infinity at $\Omega _s$ (gray dashed line). Even though the squeezing strength for light in the laboratory is rapidly being advanced^56^, we consider cases when we want to measure external forces at frequencies near $\Omega _s$ to be outside the scope of this manuscript in addition to the extreme cases mentioned before, i.e., dc field external forces and forces oscillating at relatively high frequencies with respect to system parameters. When the detection frequency is close to $\Omega _s$ the amplitude of the quantum fluctuations is much larger than the relevant classical expectation values violating our initial assumptions.

**Figure 7:**
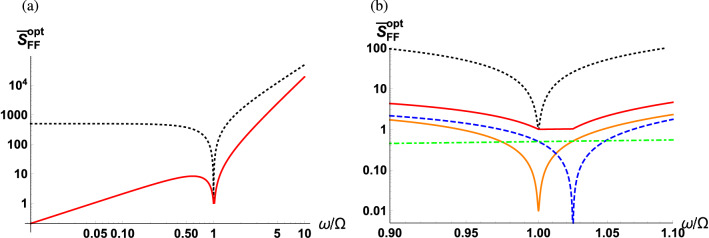
Optimum sPSD of ${\hat{F}}(\omega )$ as a function of scaled detection frequency $\omega /\Omega$ (red solid curve) along with the SQL (black dashed curve) (**a**) across the low frequency regime and (**b**) enlarged in the vicinity of the mechanical resonance. The contribution from the vacuum fluctuations (orange solid curve), the squeezed vacuum fluctuations (blue dashed curve), and the thermal fluctuations (green dot-dashed curve). The parameters used are $\kappa /\Omega = 10^2$ and $\Gamma /\Omega = 10^{-3}$.

Figure 7a shows the optimum sPSD of ${\hat{F}}(\omega )$ as a function of the scaled detection frequency for fixed optical and mechanical linewidths. The SQL is marked by a black dashed curve, and Fig. 7b illustrates a more detailed plot in the vicinity of the mechanical resonance with added curves representing contributions from vacuum fluctuations (orange solid curve) and squeezed vacuum fluctuations (blue dashed curve). When $\omega =\Omega$, the optimum sPSD becomes

45 $$\begin{aligned} \bar{S}_{FF}^{\mathrm{opt}}(\Omega ) = \frac{\Omega /2}{\sqrt{\Omega ^2+\kappa ^2/4}} +\frac{\kappa ^2/8}{\Omega ^2+\kappa ^2/4}+\frac{1}{2}. \end{aligned}$$

In the regime where $\Omega \ll \kappa$, the contribution from the squeezed vacuum fluctuations becomes dominant, resulting in $\bar{S}_{FF}^{\mathrm{opt}}(\Omega )\approx 1+\Omega /\kappa$. Therefore, no added benefits will be observed by utilizing the quantum correlations of the squeezed vacuum with quantum interference when on resonance. However, for the case of off-resonance, a significant suppression of the noise can be observed when utilizing squeezed vacuum fluctuations in the balanced MZI scheme. It is also found that the optimum sPSD of ${\hat{F}}(\omega )$ is flat across a frequency window of $\Omega<\omega <\Omega _s$ sustaining its minimum SQL value. This is well illustrated in Fig. 7b where the contribution from the vacuum fluctuations (orange solid curve) increases with the detection frequency on the blue side of the mechanical resonance, while the contribution from the squeezed vacuum fluctuations (blue dashed curve) decreases with detection frequency until reaching $\Omega _s$. Indeed the two competing contributions are generally not exactly symmetric within this frequency window implying that the sPSD might not be completely flat with zero slope. However, when the contribution from thermal fluctuations (green dot-dashed curve) is added, the sPSD becomes perfectly flat at a constant minimum value. In practice, although the observation of the flat sPSD near $\Omega _s$ is difficult even with state-of-the-art technology, the flat sPSD near $\Omega$ can be observed^56^. Note that the width of the flat sPSD window is determined by the product of the optical and mechanical linewidths, see Eq. (40).

**Figure 8:**
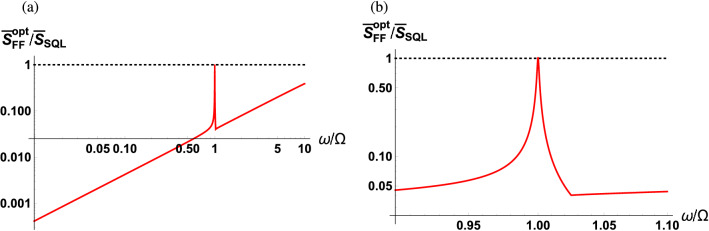
Optimum sPSD of ${\hat{F}}(\omega )$ normalized with the SQL as a function of scaled detection frequency $\omega /\Omega$ (**a**) across the low frequency regime and (**b**) enlarged in the vicinity of the mechanical resonance. The parameters used are $\kappa /\Omega = 10^2$ and $\Gamma /\Omega = 10^{-3}$.

Finally, in order to highlight the benefit of using the balanced MZI scheme, we present Fig. 8 showing the optimum sPSD normalized to the SQL. Recall that in the case where we use a classical LO field, the sPSD can be optimized to half of the SQL when off-resonance. On the other hand, as shown in Fig. 8, the sPSD can be optimized to be suppressed by multiple orders of magnitude below the SQL when off-resonance. In the later case, destructive interference engineers the photon counting noise in the balanced detection signal where its effect manifests itself in the regime when the detection frequency is much smaller than the cavity linewidth.

## Conclusions

In summary, we have explored the quantum noise introduced to the force measurements based on a balanced MZI and standard optomechanical cavity. We have employed the coherent superposition of coherent light and squeezed vacuum field to enhance the measurement sensitivity beyond the SQL. The reference field destructively interferes with the output field of the optomechanical cavity so that the photon counting noise is suppressed in the low frequency regime. We analytically find the input parameters for which the sPSD of the detected noise is minimized below the SQL. The optimal parameters include the measurement strength, squeezing direction, and squeezing strength. The force detection scheme based on a balanced MZI introduced in this manuscript shows better sensitivity compared to that based on a balanced homodyne detection with a local oscillator field in the low frequency regime. The sensitivity of the optomechanical sensor introduced in this study is enhanced by quantum correlations in squeezed states of light as well as coherent quantum-noise cancellation developed in QND measurements. These results show the potential gains in sensitivity of MZI based optomechanical quantum sensors for a variety of weak forces and fields such as Casimir force, gravitational waves, and magnetic fields.

## Methods

### Symmetrized power spectral density

This section presents the details of the procedure for obtaining Eq. (22). In order to calculate the self-PSD of the quantum noise introduced in the force measurement scheme, one needs the correlation functions for the vacuum field, the squeezed vacuum field, and the thermal fluctuations of the mechanics in the frequency domain. We first begin with the two-time correlation functions in the rotating frame at frequency $\omega _L$ for the squeezed vacuum field of strength of the squeezing *r* and squeezing direction $\theta$^57^

46a $$\begin{aligned} \langle {\hat{d}}_{\mathrm{sq}}(t){\hat{d}}_{\mathrm{sq}}(t')\rangle&= M^*\delta (t-t'), \end{aligned}$$

46b $$\begin{aligned} \langle {\hat{d}}_{\mathrm{sq}}^\dag (t){\hat{d}}_{\mathrm{sq}}^\dag (t')\rangle&=M\delta (t-t'), \end{aligned}$$

46c $$\begin{aligned} \langle {\hat{d}}_{\mathrm{sq}}^\dag (t){\hat{d}}_{\mathrm{sq}}(t')\rangle&=N\delta (t-t'), \end{aligned}$$

46d $$\begin{aligned} \langle {\hat{d}}_{\mathrm{sq}}(t){\hat{d}}_{\mathrm{sq}}^\dag (t')\rangle&= (N+1)\delta (t-t'), \end{aligned}$$

where $M =\cosh (r)\sinh (r)e^{i\theta }$, $N=\sinh ^2(r)$. Transforming the two-time correlation functions into the frequency domain by Eq. (6), we have

47a $$\begin{aligned} \langle {\hat{d}}_{\mathrm{sq}}(\omega ){\hat{d}}_{\mathrm{sq}}(\omega ')\rangle&= M^*\delta (\omega +\omega '), \end{aligned}$$

47b $$\begin{aligned} \langle {\hat{d}}_{\mathrm{sq}}^\dag (\omega ){\hat{d}}_{\mathrm{sq}}^\dag (\omega ')\rangle&=M\delta (\omega +\omega '), \end{aligned}$$

47c $$\begin{aligned} \langle {\hat{d}}_{\mathrm{sq}}^\dag (\omega ){\hat{d}}_{\mathrm{sq}}(\omega ')\rangle&= N\delta (\omega -\omega '), \end{aligned}$$

47d $$\begin{aligned} \langle {\hat{d}}_{\mathrm{sq}}(\omega ){\hat{d}}_{\mathrm{sq}}^\dag (\omega ')\rangle&= (N+1)\delta (\omega -\omega '). \end{aligned}$$

Interchanging $\omega$ and $\omega '$ and using the even parity of the delta-function, we have the correlation functions for quantum fluctuations in the quadrature operators for the field, ${\hat{X}}_{\mathrm{sq}}(\omega )= \frac{1}{\sqrt{2}}({\hat{d}}_{\mathrm{sq}}^\dag (-\omega )+{\hat{d}}_{\mathrm{sq}}(\omega ))$ and ${\hat{Y}}_{\mathrm{sq}}(\omega )= \frac{i}{\sqrt{2}}({\hat{d}}_{\mathrm{sq}}^\dag (-\omega )-{\hat{d}}_{\mathrm{sq}}(\omega ))$, in the frequency domain as

48a $$\begin{aligned} \langle {\hat{X}}_{\mathrm{sq}}^\dag (-\omega '){\hat{X}}_{\mathrm{sq}}(\omega )\rangle&= \frac{1}{2}\left[ \cosh (2r)+\sinh (2r)\cos \theta \right] \delta (\omega +\omega '), \end{aligned}$$

48b $$\begin{aligned} \langle {\hat{X}}_{\mathrm{sq}}^\dag (-\omega '){\hat{Y}}_{\mathrm{sq}}(\omega )\rangle&= \frac{1}{2}\left[ i-\sinh (2r)\sin \theta \right] \delta (\omega +\omega '), \end{aligned}$$

48c $$\begin{aligned} \langle {\hat{Y}}_{\mathrm{sq}}^\dag (-\omega '){\hat{X}}_{\mathrm{sq}}(\omega )\rangle&= \frac{1}{2}\left[ -i-\sinh (2r)\sin \theta \right] \delta (\omega +\omega '), \end{aligned}$$

48d $$\begin{aligned} \langle {\hat{Y}}_{\mathrm{sq}}^\dag (-\omega '){\hat{Y}}_{\mathrm{sq}}(\omega )\rangle&= \frac{1}{2}\left[ \cosh (2r)-\sinh (2r)\cos \theta \right] \delta (\omega +\omega '). \end{aligned}$$

Therefore, the associated PSDs defined by $S_{O O'}(\omega )=\int _{-\infty }^{\infty } d\omega '\langle {\hat{O}}^\dag (-\omega '){\hat{O}}'(\omega )\rangle$ for the squeezed vacuum field can be obtained as

49a $$\begin{aligned} S_{X_{\mathrm{sq}}X_{\mathrm{sq}}}(\omega )&= \frac{1}{2}\left[ \cosh (2r)+\sinh (2r)\cos \theta \right] , \end{aligned}$$

49b $$\begin{aligned} S_{Y_{\mathrm{sq}}Y_{\mathrm{sq}}}(\omega )&=\frac{1}{2}\left[ \cosh (2r)-\sinh (2r)\cos \theta \right] , \end{aligned}$$

49c $$\begin{aligned} S_{X_{\mathrm{sq}}Y_{\mathrm{sq}}}(\omega )&= \frac{1}{2}\left[ i-\sinh (2r)\sin \theta \right] , \end{aligned}$$

49d $$\begin{aligned} S_{Y_{\mathrm{sq}}X_{\mathrm{sq}}}(\omega )&=\frac{1}{2}\left[ -i-\sinh (2r)\sin \theta \right] . \end{aligned}$$

where $S_{X_{\mathrm{sq}}X_{\mathrm{sq}}}$ and $S_{Y_{\mathrm{sq}}Y_{\mathrm{sq}}}$ are the self-PSDs of the amplitude and phase quadrature operators, respectively. $S_{X_{\mathrm{sq}}Y_{\mathrm{sq}}}$ and $S_{Y_{\mathrm{sq}}X_{\mathrm{sq}}}$ are the cross-PSDs between the two quadrature operators. As expected, the PSDs are independent of frequency since the associated two-time correlation functions are assumed to be delta-correlated. Furthermore, one can find the PSD of the vacuum field if the squeezing strength is zero and thus the associated PSDs for the vacuum field read

50 $$\begin{aligned} S_{X_{\mathrm{v}}X_{\mathrm{v}}}(\omega ) = S_{Y_{\mathrm{v}}Y_{\mathrm{v}}}(\omega ) =\frac{1}{2}, \quad S_{X_{\mathrm{v}}Y_{\mathrm{v}}}(\omega ) = -S_{Y_{\mathrm{v}}X_{\mathrm{v}}}(\omega ) =\frac{i}{2}. \end{aligned}$$

Finally, if the two-time correlation function for ${\hat{F}}_{\mathrm{th}}$ is^58^

51 $$\begin{aligned} \langle {\hat{F}}_{\mathrm{th}}(t){\hat{F}}_{\mathrm{th}}(t')\rangle = \frac{1}{4\pi \Omega }\int _{-\infty }^{\infty }d\omega ~e^{-\omega (t-t')}\omega [1+{\bar{n}}_{\mathrm{th}}(\omega )], \end{aligned}$$

where ${\bar{n}}_{\mathrm{th}}(\omega )$ is the mean phonon occupation number in thermal equilibrium, one can easily find the PSD of ${\hat{F}}_{\mathrm{th}}$ in a similar way presented above. The PSDs of the thermal noise read

52 $$\begin{aligned} S_{F_{\mathrm{th}}F_{\mathrm{th}}}(-\omega )=\frac{\omega }{\Omega }{\bar{n}}_{\mathrm{th}}(\omega ), \quad S_{F_{\mathrm{th}}F_{\mathrm{th}}}(\omega )=\frac{\omega }{\Omega }[{\bar{n}}_{\mathrm{th}}(\omega )+1]. \end{aligned}$$

When ${\hat{F}}(\omega )$ is written as

53 $$\begin{aligned} {\hat{F}}(\omega ) = c_{Y_{\mathrm{v}}}{\hat{Y}}_{\mathrm{v}}(\omega )+c_{X_{\mathrm{sq}}}{\hat{X}}_{\mathrm{sq}}(\omega )+c_{X_{\mathrm{v}}}{\hat{X}}_{\mathrm{v}}(\omega )+c_{Y_{\mathrm{sq}}}{\hat{Y}}_{\mathrm{sq}}(\omega )+ {\hat{F}}_{\mathrm{th}}(\omega ), \end{aligned}$$

The self-PSD of ${\hat{F}}(\omega )$ can be obtained as

54 $$\begin{aligned} \begin{aligned} S_{FF}(\omega )&=|c_{X_{\mathrm{v}}}|^2 S_{X_{\mathrm{v}}X_{\mathrm{v}}}(\omega ) +|c_{Y_{\mathrm{v}}}|^2 S_{Y_{\mathrm{v}}Y_{\mathrm{v}}}(\omega ) +c_{Y_{\mathrm{v}}}^*c_{X_{\mathrm{v}}}S_{Y_{\mathrm{v}}X_{\mathrm{v}}}(\omega ) +c_{X_{\mathrm{v}}}^*c_{Y_{\mathrm{v}}}S_{X_{\mathrm{v}}Y_{\mathrm{v}}}(\omega ) \\&\quad +|c_{X_{\mathrm{sq}}}|^2 S_{X_{\mathrm{sq}}X_{\mathrm{sq}}}(\omega ) +|c_{Y_{\mathrm{sq}}}|^2 S_{Y_{\mathrm{sq}}Y_{\mathrm{sq}}}(\omega ) +c_{X_{\mathrm{sq}}}^*c_{Y_{\mathrm{sq}}}S_{X_{\mathrm{sq}}Y_{\mathrm{sq}}}(\omega ) +c_{Y_{\mathrm{sq}}}^*c_{X_{\mathrm{sq}}}S_{Y_{\mathrm{sq}}X_{\mathrm{sq}}}(\omega ) +S_{F_{\mathrm{th}}F_{\mathrm{th}}}(\omega ), \end{aligned} \end{aligned}$$

where we have assumed that the vacuum, the squeezed vacuum, and thermal fluctuations are uncorrelated to each other and have used Eqs. (49a), (49b), (49c), (49d), (50), and (52). The sPSD relevant to the homodyne detection reads

55 $$\begin{aligned} \begin{aligned} \bar{S}_{FF}(\omega )&= \frac{1}{2}\left[ |c_{X_{\mathrm{v}}}|^2+|c_{Y_{\mathrm{v}}}|^2\right] +\frac{1}{2} \cosh (2r)\left[ |c_{X_{\mathrm{sq}}}|^2+|c_{Y_{\mathrm{sq}}}|^2 \right] \\&\quad +\frac{1}{2}\sinh (2r)\left[ \cos \theta (|c_{X_{\mathrm{sq}}}|^2-|c_{Y_{\mathrm{sq}}}|^2) -\sin \theta \mathrm{Re}[2c_{X_{\mathrm{sq}}}c_{Y_{\mathrm{sq}}}^*]\right] \\&\quad +\frac{\omega }{\Omega }\left[ {\bar{n}}_{\mathrm{th}}(\omega )+\frac{1}{2}\right] . \end{aligned} \end{aligned}$$

### Optimization of $\bar{S}_{\mathrm{sq}}(\omega )$

It should be kept in mind for Eq. (24) that the sPSD stemming from the squeezed vacuum $\bar{S}_{\mathrm{sq}}(\omega )$ relies on the measurement strength, squeezing strength as well as direction. Since $\bar{S}_{\mathrm{sq}}(\omega )$ has a global minimum with respect to each of the independent parameters (*G*, *r*, and $\theta$), the order of optimization procedure does not matter. However, the degree of complication regarding the calculation depends significantly on the order. We choose to first minimize $\bar{S}_{\mathrm{sq}}(\omega )$ with respect to the angle of squeezing. The terms in the bracket in the second term can be minimized with respect to the angle of squeezing as

56 $$\begin{aligned} \left[ \cos \theta (|c_{X_{\mathrm{sq}}}|^2-|c_{Y_{\mathrm{sq}}}|^2)-\sin \theta \mathrm{Re}[2c_{X_{\mathrm{sq}}}c_{Y_{\mathrm{sq}}^*}] \right] _{\mathrm{min}}=-\left| c_{X_{\mathrm{sq}}}^2+c_{Y_{\mathrm{sq}}}^2\right| , \end{aligned}$$

and the optimum direction of squeezing is

57 $$\begin{aligned} \theta _{\mathrm{opt}} = \cos ^{-1}\left[ \frac{-|c_{X_{\mathrm{sq}}}|^2+|c_{Y_{\mathrm{sq}}}|^2}{\left| c_{X_{\mathrm{sq}}}^2+c_{Y_{\mathrm{sq}}}^2 \right| }\right] . \end{aligned}$$

Here, we have used the fact that

58 $$\begin{aligned} a\cos \theta -b\sin \theta \ge -\sqrt{a^2+b^2}, \end{aligned}$$

where

59 $$\begin{aligned} a = |c_{X_{\mathrm{sq}}}|^2-|c_{Y_{\mathrm{sq}}}|^2, \quad b =\mathrm{Re}[2c_{X_{\mathrm{sq}}}c_{Y_{\mathrm{sq}}}^*]. \end{aligned}$$

The resulting sPSD due to the squeezed vacuum fluctuations at the optimum direction of squeezing depends on the squeezing strength and the measurement strength and is thus given by

60 $$\begin{aligned} \bar{S}_{\mathrm{sq}}(\omega )\bigg |_{\theta _{\mathrm{opt}}} = \frac{1}{2} \cosh (2r)\left[ |c_{X_{\mathrm{sq}}}|^2+|c_{Y_{\mathrm{sq}}}|^2 \right] -\frac{1}{2}\sinh (2r)\left| c_{X_{\mathrm{sq}}}^2+c_{Y_{\mathrm{sq}}}^2\right| , \end{aligned}$$

where it follows from the minus sign of the second term in Eq. (60) that the contribution from the quantum correlation behaves in the opposite way to those of the photon counting and radiation pressure backaction noise. Therefore, the sPSD owing to the squeezed vacuum fluctuations can further be minimized with respect to the squeezing strength as

61 $$\begin{aligned} \bar{S}_{\mathrm{sq}}(\omega )\bigg |_{r_{\mathrm{opt}}, \theta _{\mathrm{opt}}} = \left| \mathrm{Im}[c_{X_{\mathrm{sq}}}c_{Y_{\mathrm{sq}}}^*]\right| \end{aligned}$$

at the optimum squeezing strength

62 $$\begin{aligned} r_{\mathrm{opt}} =\frac{1}{2}\cosh ^{-1}\left[ \frac{|c_{X_{\mathrm{sq}}}|^2+|c_{Y_{\mathrm{sq}}}|^2}{2\left| \mathrm{Im}[c_{X_{\mathrm{sq}}}c_{Y_{\mathrm{sq}}}^*]\right| }\right] . \end{aligned}$$

Here, we have used the fact that the inequality

63 $$\begin{aligned} a' \cosh (2r)-b'\sinh (2r)\ge \sqrt{a^{'2}-b^{'2}}, \end{aligned}$$

holds if $a'>b'\ge 0$, where

64 $$\begin{aligned} a' = \frac{1}{2}\left[ |c_{X_{\mathrm{sq}}}|^2+|c_{Y_{\mathrm{sq}}}|^2\right] , \quad b' =\frac{1}{2}\left| c_{Y_{\mathrm{v}}}^2+c_{Y_{\mathrm{sq}}}^2\right| . \end{aligned}$$

and the condition $a'>b'$ is guaranteed by the triangle inequality. Notice that the sPSD from the squeezed vacuum fluctuations at $r_{\mathrm{opt}}$ and $\theta _{\mathrm{opt}}$ is independent of the measurement strength since the fluctuations in the amplitude and phase quadrature of the squeezed vacuum depends on the measurement strength in the opposite way, see Eqs. (19) and (37). The resulting sPSD can therefore be written as

65 $$\begin{aligned} \bar{S}_{FF}(\omega ) =\frac{1}{2}\left[ |c_{X_{\mathrm{v}}}|^2+|c_{Y_{\mathrm{v}}}|^2\right] +\left| \mathrm{Im}[c_{X_{\mathrm{sq}}}c_{Y_{\mathrm{sq}}}^*]\right| +\frac{\omega }{\Omega }\left[ {\bar{n}}_{\mathrm{th}}(\Omega )+\frac{1}{2}\right] . \end{aligned}$$
